# Alpha NSW: What would it take to create a state-wide paediatric population-level learning health system?

**DOI:** 10.1177/18333583231176597

**Published:** 2023-07-07

**Authors:** Michael Hodgins, Nora Samir, Susan Woolfenden, Nan Hu, Francisco Schneuer, Natasha Nassar, Raghu Lingam

**Affiliations:** 1 University of New South Wales, Australia; 2 Sydney Institute for Women, Children and their Families, Sydney Local Health District, Australia; 3 Child Population and Translational Health Research, The University of Sydney, Australia; 4 Community Child Health, Randwick, The Sydney Children’s Hospitals Network, Australia

**Keywords:** child health, data systems, learning health system, systems integration, health information management

## Abstract

**Background:**

The health and well-being of children in the first 2000 days has a lasting effect on educational achievement and long-term chronic disease in later life. However, the lack of integration between high-quality data, analytic capacity and timely health improvement initiatives means practitioners, service leaders and policymakers cannot use data effectively to plan and evaluate early intervention services and monitor high-level health outcomes.

**Objective:**

Our exploratory study aimed to develop an in-depth understanding of the system and clinical requirements of a state-wide paediatric learning health system (LHS) that uses routinely collected data to not only identify where the inequities and variation in care are, but also to also inform service development and delivery where it is needed most.

**Method:**

Our approach included reviewing exemplars of how administrative data are used in Australia; consulting with clinical, policy and data stakeholders to determine their needs for a child health LHS; mapping the existing data points collected across the first 2000 days of a child’s life and geospatially locating patterns of key indicators for child health needs.

**Results:**

Our study identified the indicators that are available and accessible to inform service delivery and demonstrated the potential of using routinely collected administrative data to identify the gap between health needs and service availability.

**Conclusion:**

We recommend improving data collection, accessibility and integration to establish a state-wide LHS, whereby there is a streamlined process for data cleaning, analysis and visualisation to help identify populations in need in a timely manner.

## Introduction

Despite the noted importance of health and development of children in the first 2000 days and its impact on their educational achievement and risk of chronic disease in later life (Walker et al., 2011), 20–25% of Australian children are developmentally vulnerable on school entry. This has consequences across clinical, social, education, research sectors, and state and national policy, with marked variability between social, economic and ethnic groups (AEDC, 2020). Current healthcare models provided in the first 2000 days compound this inequity by being fragmented and complex (Braithwaite et al., 2015), with unwarranted variation in access, quality, cost and outcomes (Braithwaite et al., 2018). To improve the health of all Australians, we need to intervene earlier to optimise the life chances of children throughout the life course, deliver better value health care and optimise hospital use to where it is needed most (Jones et al., 2018).

The past two decades have seen an unprecedented revolution of medical data digitalisation across major clinical settings (Hambleton and Aloizos, 2019). The large volume of electronic medical records (EMRs) collected routinely on each occasion of health service delivery has greatly strengthened the data infrastructure for informing evidence-based clinical decisions, intervention strategies and policies in a more effective and timely manner. The definition of learning health system (LHS) first proposed by the US Institute of Medicine (2007) is the process to synthesise routinely collected electronic data including EMRs into novel evidence that can improve clinical decision-making, enhance the predictive accuracy of a health condition or better inform the development of a safer and more effective treatment and intervention regime. This definition has been critiqued for “leaving out the details that would guide healthcare organizations on the specific work they should be carrying out” (Easterling et al., 2022: 2). More recent definitions have described LHSs as enabling “continuously and rapidly operating virtuous cycles of study, feedback, and practice change, regardless of the original intention for data collection” (Platt et al., 2020: 2) and “a system in which routine health practice data, from service delivery and patient care, can lead to iterative cycles of knowledge generation and healthcare improvement” (Enticott et al., 2021: 2).

Gardner and Kelleher (2014) have proposed characteristics that a paediatric LHS would possess that distinguish it from an adult LHS. First, a paediatric LHS should emphasise how to prevent the onset of chronic conditions and promote early development and well-being of children. Second, a paediatric LHS should have the capacity to measure and track childhood development over time to understand qualities that promote healthy child, adolescent and subsequent adult life. Third, a paediatric LHS must expand its mission from traditional health care to community health by integrating the health system with schools, social service agencies and other institutions that interact with children and families (Gardner and Kelleher, 2014). These distinct features for a paediatric LHS, based on the social determinants of child health and the importance of multidisciplinary teams, provide important guidance for the development of an LHS.

To inform services, we need population-level data to understand how children are progressing through our health systems, their health status and the impact of the care provided. The First 2000 Days Framework is a New South Wales (NSW) initiative aimed at ensuring children in NSW have the best outcomes in the first 2000 days of their life. The NSW First 2000 Days Framework selected core health and development indicators in the early years of life including maternal factors during the perinatal period, neonatal development, hospitalisations and emergency department (ED) attendances due to common causes for children, and school readiness outcomes. These indicators cover a range of health service settings (primary, secondary and tertiary care), community services and education. The range of service settings makes integration and targeting of services extremely difficult due to separate data silos. Data linkage is solving some of these issues but not necessarily in a timely way.

Within health, data are also divided between agencies and health providers. In addition, there is a lack of integration between high-quality data, analytic capacity and timely health improvement initiatives. Practitioners, service leaders and policymakers cannot use available data effectively to plan and evaluate early intervention services and monitor high-level health outcomes. This disconnect between data and health system change is what the Alpha NSW project aimed to address. Our exploratory study aimed to develop an in-depth understanding of the system and clinical requirements of a state-wide paediatric LHS that uses routinely collected data to not only identify where the inequities and variation in care are, but also to inform service development and delivery where it is needed most.

## Method

The project was comprised of four phases: *Phase 1* involved reviewing two Australian case examples of the use of administrative data for LHS, including the Western Australian Child Development Atlas (WACDA) and the Children’s Health Queensland (CHQ) dashboard. This review explored the aims of each LHS, the child health indicators included, the data sources and the geospatial presentation created as part of the LHS.

*Phase 2* involved a series of focus groups and one-to-one interviews with 40 clinical, policy and data stakeholders across participating NSW sites to determine their needs for an NSW paediatric LHS. The focus groups and interviews were semi-structured, with questions aimed to guide a discussion of data needs for clinicians during their care of children in the first 2000 days, and barriers and facilitators to accessing data to improve practice. Verbatim transcripts of interviews were analysed thematically following Braun and Clarke’s (2006) approach.

*Phase 3* involved mapping the various data sources collected across the first 2000 days of a child’s life to determine what data are available and accessible to inform service delivery. Data sources, formats, storage and information governance procedures for key health variables in the first 2000 days of the child’s life in NSW were collated from a key document search of data points from health, community and education along with targeted services such as sustained nurse home visiting. The data table was shared with key informants as part of their service role to ensure completeness.

*Phase 4* involved the use of the Kids-Link research data to produce geospatial maps for key indicators for child health needs. This was to demonstrate the potential of using routinely collected administrative data to identify the gap between health needs and service availability. Our analyses were based on the linkage of NSW administrative health data, including the NSW Admitted Patient Data Collection, which comprises all hospital inpatient records from both private and public hospitals, NSW Emergency Department Data Collection, of all ED attendances in public hospitals, the NSW Perinatal Data Collection comprising all births in NSW and the Australian version of the Early Development Instrument (AvEDI), a nationwide teacher-based assessment of child development outcomes and school readiness conducted every 3 years. Relevant Socio-Economic Indexes for Areas (SEIFA) scores were applied for each local government area, statistical areas level 2 (SA2) and then categorised into quintiles ranging from most disadvantaged (quintile 1) to least disadvantaged area (quintile 5). Similarly, the Accessibility/Remoteness Index of Australia (ARIA+, 2022) was applied to each SA2 to determine metropolitan, regional, rural or remoteness of area. Using mapping software, these data were then overlaid to corresponding maps of NSW to enable geospatial analysis and presentation of the patterns and distribution of indicators (see Supplemental files for maps). These data were linked by the NSW Centre for Health Record Linkage and approval for the use and release of deidentified data was provided by the NSW Population and Health Services Research Ethics Committee (2019/ETH11532).

Findings from these four phases were synthesised to create recommendations for future research and policy. The project received ethics approval from the Sydney Children’s Hospitals Network Human Research Ethics Committee on 3 September 2019 (ETH12039). An amendment for the ethics application was also approved on 1 July 2020.

## Results

### How have child health data been used effectively in Australia? (Phase 1)

Two comprehensive examples of tools using administrative data to inform place-based intervention strategies and policies were identified in two Australian states. These cases exemplify the potential of population-level LHS where routinely collected health data can be used to evaluate the geospatial distribution (patterns) of population health outcomes and health service availability and accessibility.

#### Western Australian Child Development Atlas

The WACDA project uses state-wide routinely collected data from multiple government agencies to create the community profiles of child development, health and social outcomes by aggregating area-level data (https://childatlas.telethonkids.org.au/) (Fuller et al., 2020). These spatial patterns of child outcomes are overlaid by locations and catchment areas of child and family-oriented services and programs across the state by using geographic information system. This atlas reveals areas of high need that do not have adequate or appropriate services. The WACDA serves as a spatial data visualisation tool that can facilitate inter-agency collaborations involving policymakers, service providers and communities to improve monitoring of trends and inform the place-based approaches to planning, development and delivery of policies, services and programs aimed to address complex problems confronting children and families in areas of greatest need. An example of the types of maps is given in Supplemental Figure S3.

The WACDA is able to create a wide range of area-level indicators for child health and development outcomes, using routinely collected data by different government agencies responsible for various services including health (admitted patient care, ED), child and adolescent mental health, maternal and child health, early childhood and school education, disability support, drug and alcohol support and juvenile justice and the Australian Bureau of Statistics (ABS, 2016) (see Supplemental Box S1 for the specific indicators). These indicators are geographically mapped against the ABS standard geographic regions SA2 and SA3 and other administrative geographic boundaries (e.g. child protection districts, electoral divisions) to enable the visualisation and exploration of the relationships between child health and development indicators and place-based and socio-economic characteristics.

#### Children’s Health Queensland population health intelligence dashboard

CHQ has developed a population health intelligence dashboard that brings together more than 35 datasets from multiple government agencies to profile the health and well-being of children at a population level (Callard and Tracey, 2021). This dashboard is aimed to support more informed health services planning and to ensure efforts are focused on communities that need them most. In addition, by integrating data from different government sources, CHQ aims to take a holistic approach to understanding child development at different stages. This would not only help people to identify the best time to intervene to achieve better outcomes for children, but also to enable the prediction of the social and economic outcomes for children in the future.

The CHQ Children of Queensland Indicators Framework, which incorporates a range of national and international evidence-based indicators, guided the development of the dashboard (Supplemental Box S1). These indicators can be extracted from records routinely collected by different government agencies and presented against different areas or regions using data visualisation techniques to help identify communities in greater need of services more effectively. For example, Supplemental Figure S2 shows the regional proportion of children assessed to be developmentally vulnerable at school entry age using the AvEDI data by the levels of neighbourhood socio-economic status (SES) measured using the SEIFA. It not only captures the pattern of association between developmentally vulnerable and neighbourhood SES but also identifies specific regions where children are mostly affected by developmental vulnerability. Another example (Supplemental Figure S3) graphically reveals the positive correlation between two health indicators: low birthweight and maternal smoking during pregnancy. Since each dot represents the location of a region by the level of each health indicator, four groups of regions can be identified. Such grouping based on the visualised pattern of health indicators by region can serve as a tool to identify regions in need of effective interventions to help women cease smoking before and during pregnancy to reduce the risk of low birthweight of the neonates, such as regions falling in the “high maternal smoking level, high low-birthweight rate” group. In addition, this visualisation helps identify not only the regions with a high maternal smoking rate but also the low level of low birthweight. Further explorations should aim to understand what protective factors or effective intervention measures take place in these regions, and if these protective factors and measures can be applied to help other regions with a high maternal smoking level to reduce the low-birthweight rate. Likewise, for regions with a low maternal smoking rate but a high rate of low birthweight, additional risk factors need to be investigated.

### What do frontline workers need for a paediatric LHS? (Phase 2)

Our formative research with 40 stakeholders aimed to understand the logistic and policy facilitators and barriers to setting up a population paediatric LHS and determine what frontline workers need from a platform to inform service development and delivery. The key themes identified from this qualitative work ranged from how to access health data and the kinds of health data clinicians would like to access, to some of the institutional and systemic changes needed to support more effective use of data in clinical settings.

#### Variation in health data collections

Our consultations with data experts in NSW yielded concerns about the complex context of NSW Health data. Not only do NSW Health EMR systems differ across the state, but the way these systems are used, and the type of data clinicians provide in these systems also differs dramatically. Variations between scanned documents, open text boxes and extractable forms exist across homogeneous healthcare services as similar kinds of data are stored in different ways in different systems. In addition, as part of the Australian context, an LHS based in NSW will need to consider crossing the public/private divide, a task identified as “not achievable” by several consultation participants. This complexity presents formidable political and practical challenges.

#### Competing demands of health data

Clinician participants identified a desire for data to inform their clinical practice in different ways. Primarily, clinician participants reported wanting data to track the trajectory of children and their families before, during and after contact (i.e. use data to evaluate client-based outcomes for their services). Clinicians wanted greater visibility across specialties by viewing data from other services to integrate care and ensure consistency of care for children in the first 2000 days. For example, child and family nurses reported wanting greater access to longitudinal individual outcomes for families, including birth and maternal history data. Allied health practitioners conversely described wanting greater visibility of patient’s trajectory pre-referral: “I want individual service linkage data to be able to capture if there is a trend in journeys through services. Do women have similar journeys through [maternal] services?” This desire for information often transcended health, as clinicians desired greater access to external service data such as child protection, legal and education data:

To be able to extract data to show that child protection reports have gone up, domestic violence reports have gone up, is good data not only for our service but broadly across the district in acute service, as well, [to show] we really need to be increasing funding in this space, we know the impact it has in the first 2000 days if we don’t respond. (Child, youth, and family services director)

Participants reported wanting to use data to target services to “pockets of vulnerability,” or gaps in service provision within their communities. As one project coordinator stated, they wanted the opportunity to “show where children are having to wait X amount of time for speech therapy, that’s a big one right across the state.” Many participants reported a desire to use data to target early intervention for vulnerable families, for instance, additional home visiting and psychological support services. One example that arose during data collection was the use of Adverse Childhood Experiences (ACEs) data to help inform service delivery:

My service is across five sites, so it would be useful to be able to compare [ACEs data] across the sites and it would be really useful to compare that data to other services as well . . . I know that a large proportion of children with speech and language difficulties early on also have social emotional flags . . . in terms of prevention, [using that data] to link my service with other services like counselling would be useful. (Speech pathologist)

Another example was the use of service access data to explore why families were disengaging from universal child and family services over time:

There is a significant drop off the older children get, and it would be very interesting to know why those families are disengaging and who is using the service . . . because that’s where we are supposed to be picking up children’s vulnerabilities early. (Child and family health nurse)

Clinical participants described seeing great value in the use of data to geographically target services and populations. Participant responses to the notion of using data to target services ranged from targeting services at an individual level and at a geographic level, although many participants identified confidentiality concerns around using individual-level data, as one child, youth and family services manager stated, “[using data to] target services at a broader population level, in regard to suburbs . . . would be really useful.” Additionally, participants wanted to compare their service provision and outcomes with other services to inform what they were doing well and what aspects of practice and outcomes could be improved. Personnel in managerial and executive positions reported a desire for data to assist regular reporting to government agencies.

#### Barriers to accessing health data

In addition to data collected about clinician’s data preferences, we also collected qualitative data about clinicians’ preferences in terms of data access. In some instances, for clinicians, the data necessary to respond to some of these needs were available, but barriers to access proved challenging. In one of the sites of qualitative data collection, clinicians had access to a dashboard built to provide population data for the area and guide service delivery. Despite having access to “thousands and thousands of lines of data” clinicians cited barriers to accessing the information inherent in the data. They had many barriers including a busy workload, limited self-efficacy in navigating a data dashboard and the narrow focus of the dashboard limiting applicability to certain services and personnel. The use of the dashboard was also hampered by incomplete data, as one community health information management unit manager noted “one child might have been weighed once and not weighed again so there’s no way to compare that child.” The concern expressed by the participant here reflected the tension that arose in discussion with participants regarding population versus individual data.

Some participants found the dashboard overwhelming because of the many different data subsets and the overall presentation, preferring “simpler” and more “straightforward” presentations of data, such as infographics. One participant commented that “you literally need a PhD to understand [the dashboard].” The nature of the dashboard given its focus on child and family nursing also narrowed its utility for the service “we’ve struggled to get people to engage with a dashboard, it is based on child and family nursing and there is not a lot for our allied health team [children and communities program coordinator].” The difficulties of accessing health data were also echoed at the second site with regional data collection. Clinician participants described not having easy access to the data they needed, and even when data were available, it was “not in a format that you can use” or not readily “relevant.” Clinicians specifically cited the inability to use data to “pinpoint services for improvement” or target services to area of need.

Managing a co-design process as part of the development of an LHS is vital to its successful uptake by clinicians, according to all consultation participants. Consultation participants identified the importance of “translators” within the development process, that is, individuals with complimentary clinical and information technology expertise and/or experience. This is a role potentially provided by health information managers, but participants in our study did not find them accessible. Some participants also noted the flaws in a deficit approach to informatics, as clinicians may feel like they are being “bashed” with data in that they are provided data that show flaws or deficits in their practice. Again, participants noted the importance of including clinicians early in the process of development to mitigate this potential risk.

#### Lack of analytical support

Across the interviews with clinical personnel, a persistent theme identified was the lack of, and subsequent need for, resources to assist with data analysis. Access to health data was often less of an issue than what to do with the data once collected. Clinicians regularly reported the need for greater analytic capacity locally. This ability to meaningfully analyse data has the potential to support the targeting of services as well as the improvement of practice. Clinicians discussed wanting to use data to create interventions and target them to specific needs, as evidenced by this child and family health nurse’s comment: “We get on the treadmill of churning out clients but that doesn’t help in future planning and service delivery . . . I don’t think we have a coordinated effort [to use data for early intervention].” Clinicians suggested greater support, in terms of resources and personnel to identify and target interventions based on the use of data, would be acceptable and largely welcomed as long as it was attentive to the local needs and context. Participants cited the value in having support to target services and implement new ways of working “as long as it is based within our service and can work with, rather than just tell us what to do.”

### What are the current child health data sources in NSW and where are these data currently held? (Phase 3)

**Figure 1.**
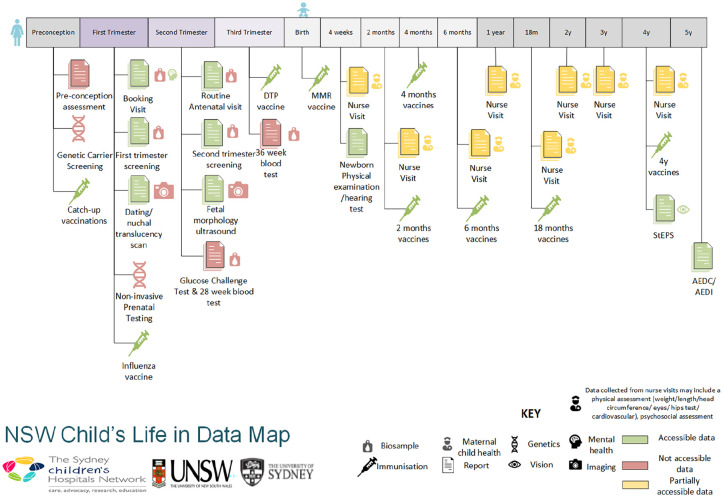
An NSW child’s life in data.

NSW Health have published a First 2000 days Priority Framework (Health NMo, 2019). A table of data collections that cover services from preconception to the end of the first 2000 days of the child’s life is included in Supplemental Table S2, and a visualisation of the points of data collection and accessibility is given in Figure 1. Moreover, half of data collections from preconception to the first 2000 days of a child’s life are not accessible to healthcare providers and researchers, largely due to the lack of integration of data across primary, secondary and tertiary care. For example, data from antenatal visits are not linked between GPs and hospitals. Furthermore, many of the indicators are owned by various custodians which may contribute to the lack of accessibility and linkage of the data.

While reviewing this framework, we also identified a number of factors related to child health and listed them as priority areas, for which NSW Health does not currently routinely collect data. These include maternal alcohol consumption and information on partners.

### What could a paediatric LHS do in NSW?

The development of a LHS that supports the First 2000 Days indicators requires an understanding of the data available for analysis and a method of analysing and presenting those data. This section provides examples of the type of indicators that can be explored using various NSW Health data collections and modes of presentation and comparison across SES and geographical areas to demonstrate the potential of using the routinely collected health records to identify current status and inform place-based interventions and policymaking across the state. A range of core health and development indicators in the early years of life were selected as per the NSW First 2000 Days Framework including maternal factors during the perinatal period, neonatal development, hospitalisations and ED attendances due to common causes for children, and school readiness outcomes (see Supplemental Table S2 for list of indicators). In this demonstration, our analyses were based on health records routinely collected by the NSW hospitals, including hospital inpatient records from both private and public hospitals, all ED attendances in public hospitals and perinatal data recorded by midwives for all births in NSW. Child development outcomes were based on data from the AvEDI.

**Figure 2.**
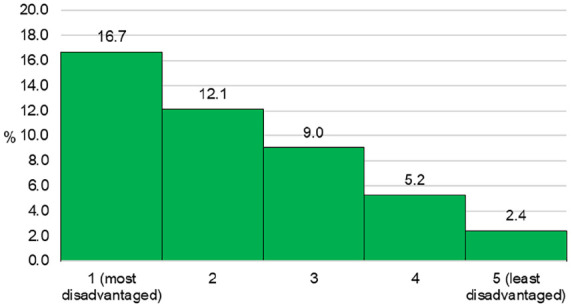
Smoking during pregnancy by levels of socio-economic disadvantage in NSW in 2019.

**Figure 3.**
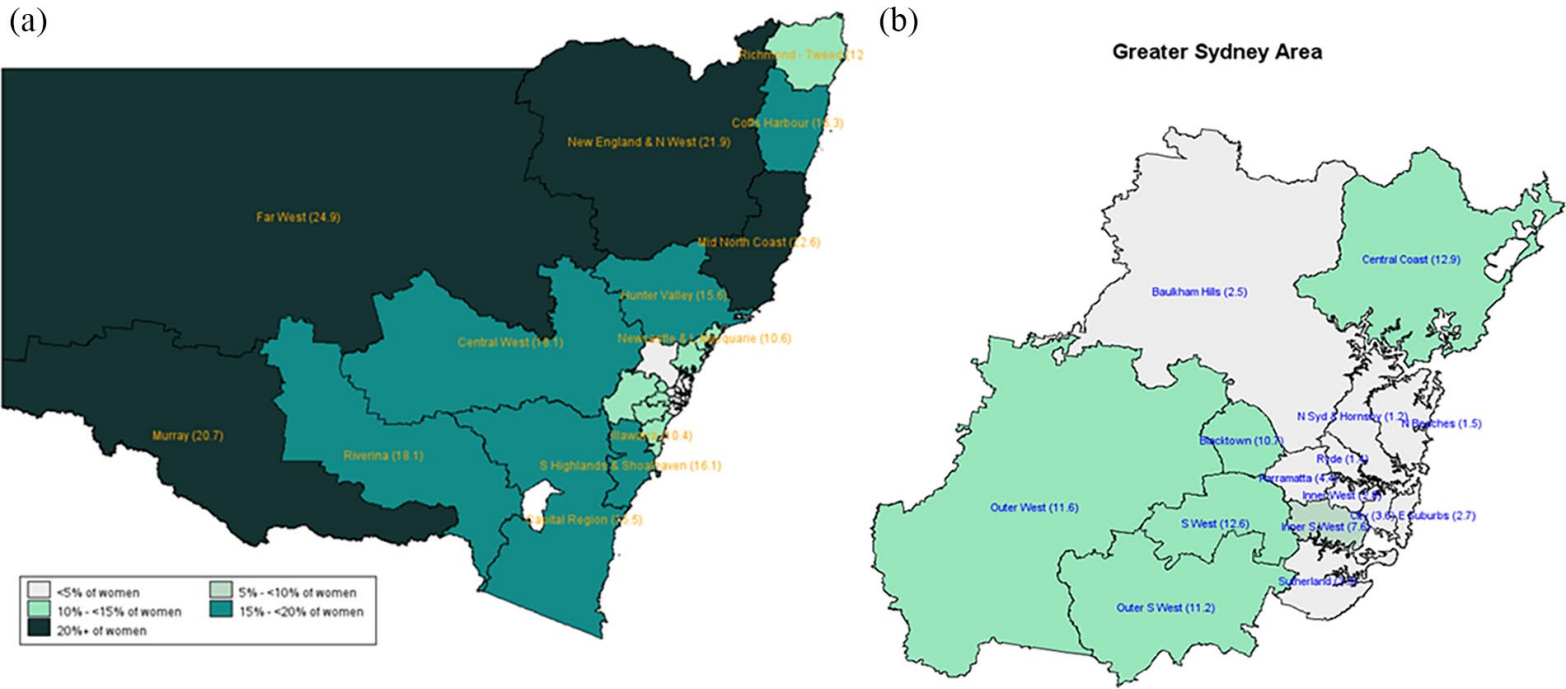
Smoking during pregnancy by SA4 in NSW in 2019.

**Figure 4.**
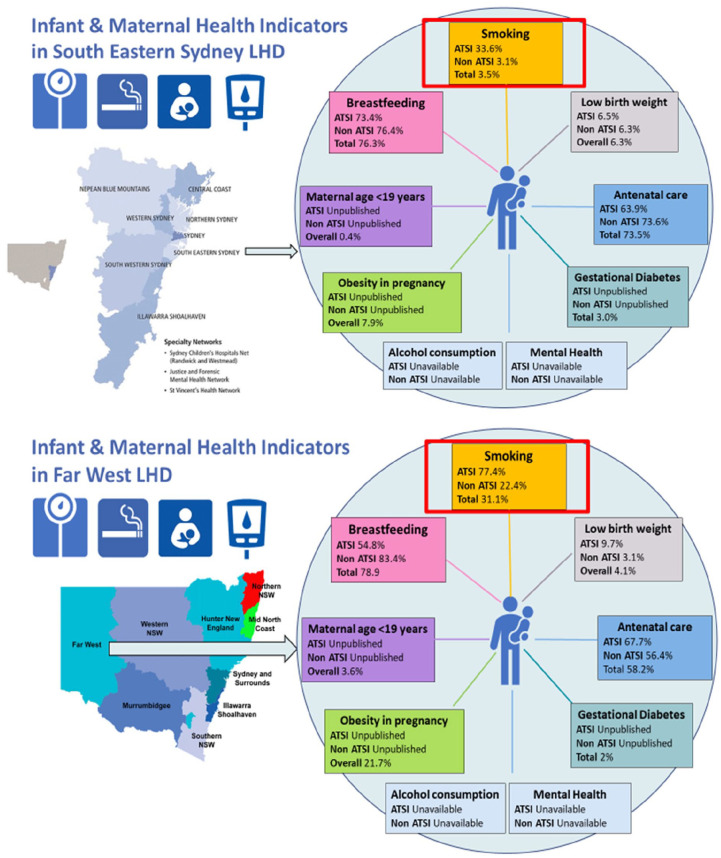
Exemplar maps for infant and maternal health indicators in different LHDs.

Information on SA2 of residence enabled the determination of SES and urban–rural location based on the national standard classification developed by the ABS. Figure 2 shows the disparity in the smoking rates among pregnant women residing in areas with different levels of socio-economic disadvantage. It shows that the prevalence of smoking was 16.7% for those living in the most disadvantaged areas, compared to only 2.4% for those from the least disadvantaged areas. Figures 3 and 4 demonstrate the more granular geospatial variation of maternal smoking rate across NSW.

Similar to what has been done in CHQ, using publicly available data from HealthStats NSW (NSW Minstry of Health), we then created a map of infant and maternal health indicators for different Local Health Districts (LHDs) around NSW to demonstrate the potential of synthesising routinely collected data to create the health and development profiles of children and families in each LHD. As shown in Figure 4, this map is more friendly for clinicians, service providers and policymakers, allowing them to not only easily compare indicators between different LHDs, but also identify needs in a particular location and develop interventions targeted to address these needs.

## Discussion

Our work has demonstrated that data to evaluate the first 2000 days indicators are dispersed between providers and service settings. We also identified the need for key clinical, policy and research stakeholders to track the health and developmental trajectory of children and their families at an individual and population level across the first 2000 days. This includes their contacts with health services, comparing child health outcomes across services, understanding their needs for better health and development outcomes and using data to develop interventions to address the needs. It is evident that the early detection of health needs across populations relies on the administrative records to be collected and integrated from different agencies for the analysis in a timely manner. The two national exemplars we have presented, the WACDA and CHQ, have demonstrated the potential to use administrative routine data collections and geospatial mapping techniques to help researchers, practitioners and policymakers to identify the health and social needs in different areas. They also highlight the critical requirements for a successful paediatric LHS including early detection of standardised indicators, longitudinal monitoring and integration of administrative data from various agencies. This requires a highly centralised system or data hub to be established. Our analysis highlighted that geospatial mapping can be used to address areas of health inequality and to serve as a powerful tool for the planning, development and implementation of local early childhood health and family services and interventions.

In NSW, the NSW Health Analytics Framework has highlighted the need to increase the use of analytics to better deliver on the purpose and direction of the health system. In Australia, the majority of the EMRs available represent tertiary health service use such as hospital inpatient admissions, ED attendances and hospital outpatient visits. State-wide primary health data (e.g. GP visits) are generally not available, although primary health data in some LHDs have been consolidated and developed for use. When considering health service use, we need to consider linkage between primary and secondary care data sources to better understand a person’s complete health service use pathway. This will also help us to implement early interventions appropriately. One way of doing this, is by accelerating the transition to EDWARD, a data platform that has been designed to replace the Health Information Exchange system to collect, manage and safely use high-quality data and information across all parts of the health system. It will act as NSW Health’s strategic data source for performance monitoring, health service planning and disease surveillance. EDWARD covers a range of data domains such as Community Health Outpatient Service Events, ED, Admitted Patient and Mental Health, allowing users to “take an information journey” from the facility, to the LHD and across NSW.

### Recommendations

**Figure 5.**
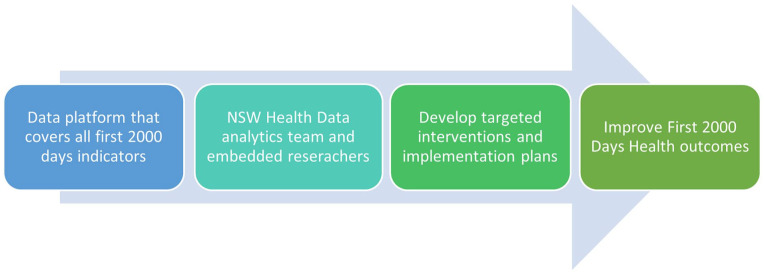
Alpha NSW framework.

To help address the existing issues and increase the use of analytics to better deliver on the purpose and direction of the health system we propose an LHS called Alpha NSW. This will build on the NSW Health Analytics Framework, by envisaging a data platform that integrates the first 2000 days’ indicators and allows health providers and policymakers to track the trajectory of children across services and develop targeted interventions that meet the population’s specific needs. This platform should be owned by NSW Health, who can control access to, and use of data by, local sites. There is great potential of using cross-sector integrated routinely collected administrative data to establish a highly effective LHS that can automatically synthesise data to produce new knowledge (e.g. identification of areas subject to poor health outcomes and insufficient health services to address the problems) that is subsequently fed back into the health system to inform the development of more functioning intervention strategies that can accurately target the needs of population in need. With the revolutionary digitalisation of medical records, NSW Health has accumulated a considerable number of records documenting the person-level health service use experiences. This has contributed significantly to the health research and the development of intervention strategies. However, it can be strengthened in the future by integrating more health programmes, surveys and service data to improve the data infrastructure to better inform early, targeted and coordinated health interventions. Furthermore, Alpha NSW should include resources and personnel to identify and target interventions. In summary, Alpha NSW (Figure 5) can be thought of not as a data platform or repository but as a functioning LHS where:

- Data covering the first 2000 days is cleaned and stored
- Analysts work with embedded statisticians based in LHDs to conduct data analyses such as the examples we have provided that address clinically important questions that are relevant to the priorities of the LHD
- Subsequently, implementation scientists and local clinicians develop targeted interventions and implementation plans and evaluate them to improve First 2000 Days Health outcomes.

One of the added benefits of this approach is that it allows both NSW Health and local sites to have oversight of data, keeping it “in house” and using functionally for system strengthening. This will lessen the concerns surrounding privacy and confidentiality of patient information that present a substantial barrier to the large-scale access and use of health information.

Our vision aligns with that of Chambers et al.’s (2016) advocacy for the inclusion of evidence-based strategies (e.g. system change interventions, training, supervision, quality monitoring tools) to improve the integration of clinical interventions within real-world practice. They envisage “repositioning the formal health care delivery sector as a set of nimble organizations that focus on ongoing system improvement by capturing data at the clinical encounter and using those data to inform ongoing clinical and community practice” (Chambers et al., 2016: 1). It is vital to focus on identifying strategies that support the adoption, implementation, sustainability and ongoing improvement of data collection and data-informed interventions. This requires the embedding of data collection within ongoing healthcare delivery settings to aggregate data; improving mechanisms for rapid referral of patients to needed services and implementing strategies to improve disease management, monitoring and clinician follow-up.

A state-wide LHS could address multiple needs. However, these needs are often at odds with one another. For instance, there is an inherent tension between retrospective data serving research and policymaking interests and real-time data needed for instantaneous, predictive, clinical decision support. From a service provision perspective, there remains a need for data that assist both the delivery and evaluation of services and align with reporting requirements expected by policymakers and governing bodies. These competing interests pose challenges in terms of determining the nature and level of data collected, for instance, individual-level data versus population-level data. McLachlan and colleagues (2018) noted that the LHS community is fragmented, with little awareness or partnership across approaches and systems. The use of existing data from NSW and examples of LHS from WA and QLD demonstrate the great potential of using routine administrative data collections and geospatial mapping techniques to help practitioners, policymakers and researchers to identify the health and social needs in different areas, particularly those characterised by higher levels of socio-economic disadvantage. The critical requirements for a successful paediatric LHS include early detections, longitudinal monitoring and integration of administrative data from various agencies. Future work should focus on emulating these in a nationwide system.

## Conclusion

We recommend improving data collection, accessibility and integration to establish a state-wide LHS, whereby there is a streamlined process for data cleaning, analysis and visualisation to help identify populations in need in a timely manner. This LHS would not only allow analysts to support NSW Health personnel to identify areas of high need, but also enable implementation scientists to develop early and targeted interventions that will ensure equitable care for all NSW children.

## Supplemental Material

sj-docx-1-him-10.1177_18333583231176597 – Supplemental material for Alpha NSW: What would it take to create a state-wide paediatric population-level learning health system? Supplemental material, sj-docx-1-him-10.1177_18333583231176597 for Alpha NSW: What would it take to create a state-wide paediatric population-level learning health system? by Michael Hodgins, Nora Samir, Susan Woolfenden, Nan Hu, Francisco Schneuer, Natasha Nassar and Raghu Lingam in Health Information Management Journal
