# Supplementary material for: Alpha NSW: What would it take to create a state-wide paediatric population-level learning health system?
Source: Health Inf Manag. 2023 Jul 7;53(3):217–26. doi: 10.1177/18333583231176597 (PMC11401336; doi:10.1177/18333583231176597)

**Alpha NSW: What would it take to create a state-wide paediatric population level learning health system?**

Supplementary files include:

- Box S1 Points of data collection from preconception to the first 2000 days of the child’s life
- Table S1 Datasets, indicators and populations used to examine patterns of maternal and child health outcomes
- Figure S1 The Western Australian Child Development Atlas example data visualisation maps
- Figure S2 Children’s Health Queensland Population Health Intelligence Dashboard example data visualisation tools
- Figure S3 Children’s Health Queensland Population Health Intelligence Dashboard example data visualisation tool

# Box S1

Points of data collection from preconception to the first 2000 days of the child’s life

**Panel 1. The Child Development Atlas indicators**

**Pregnancy and births**

- Proportion of children born low birthweight (born alive < 2,500g)
- Proportion of pre-term births (< 37 weeks and <39 weeks respectively)
- Proportion of children born to teenage mothers (15-19 years and 0-24 years respectively)

**Physical Health related hospitalisations**

- Chronic physical conditions related hospitalisations for 0-24 year olds (cancer, cardiovascular, diabetes, ear and hearing, oral, obesity, kidney, musculoskeletal, respiratory related conditions)
- Vaccine preventable disease related hospitalisations for 0-24 year olds
- Disability related hospitalisations for 0-24 year olds
- Injury related hospitalisations (intentional vs unintentional) for 0-24 year olds
- Assault related hospitalisations for 15-24 year olds
- Alcohol and drug related hospitalisations for 15-24 year olds

**Mental health related hospitalisations and ED attendance**

- Mental health related hospitalisations for 10-24 and 15-24 year olds respectively
- Mental health related ED presentations for 10-24 and 15-24 year olds respectively
- Deliberate self‐harm related hospitalisations for 10-24 and 15-24 year olds respectively
- Deliberate self‐harm related ED presentations for 10-24 and 15-24 year olds respectively
- Proportion of births to mothers with a mental illness diagnosis
- Contacts with a public community/outpatient mental health service for 0‐24 year olds

**Education**

- Children developmentally vulnerable or at risk on the Australian version of the Early Development Instrument domains
- Developmental vulnerability on 1 or more/2 or more Australian version of the Early Development Instrument domains
- Developmentally ‘on track’ on all five Australian version of the Early Development Instrument domains
- Proportion of children attending preschool for 15 hours or more
- Average National Assessment Program – Literacy and Numeracy (NAPLAN) scores in reading, writing, spelling, grammar & punctuation, and numeracy of students in Year 3, 5, 7, and 9
- Proportion of 0-24 year olds who do not speak English well or at all
- Proportion of 0-24 year olds with Year 12 or equivalent highest year of school completed

**Juvenile offence**

- Assault offenders (family and non-family) (per 10,000 persons aged 10-24 years)
- Offenders (involved in different categories including burglary, drug offence, stealing, threatening) (per 10,000 persons aged 10-24 years)

**Mortality**

- Infant mortality rate (number of deaths per 1,000 live births)
- Child mortality rate (number of deaths per 100,000 0-4 year olds)
- Suicide rate (number of deaths by suicide per 100,000 10-24 year olds)

**Socioeconomic status**

- Proportion of low-income households
- Proportion of occupied private dwellings with no internet connection
- Proportion of people unemployed
- Language other than English spoken at home

**Service Use**

- Rate of Emergency Department presentations in children and young people (per 1,000 persons)
- Contact with Ngala parenting helpline service

**Panel 2. CHQ Children of Queensland Indicators Framework**

**Living in Queensland**

- Distribution – Population served
- Projections – Population growth
- Remoteness and Deprivation

**Being loved and safe**

- Family Environment - Safe

**Having material basics**

- Family Environment – Basics
- Family Environment – Parental Behaviours

**Learning**

- Educational Indicators
- Childhood Development

**Participating**

- Community Engagement
- Spatial / Environmental Indicators
- Connected Communities
- Support Services

**Being healthy**

- Maternal and Child
- Behavioural Risk
- Risk Factors and Chronic Conditions
- Mortality

**Using health services**

- Use of Health Services - Hospitalisations
- Use of Health Services – Primary Care and Community

# Table S1

Datasets, indicators and populations used to examine patterns of maternal and child health outcomes

| Dataset | Indicators | Study population | Data presentation |
| --- | --- | --- | --- |
| NSW Perinatal Data Collection (PDC) | - maternal smoking during pregnancy - antenatal care starting after 20 weeks of gestation - pregnancy <18 years - preterm birth (<37 weeks gestation) - low birthweight (<2500g) - breastfeeding at discharge | All livebirths in NSW recorded in PDC and sub-divided by statistical areas 4 (SA4) ^1^ | The level for each indicator was presented by   - Australian Bureau of Statistics (ABS) Socioeconomic Indexes for Areas disadvantage quintiles - statistical areas 4 (SA4) |
| Emergency Department Data Collection (EDDC) | - rate of ED presentation   calculated as the number of all ED presentations (the numerator) being divided by the number of children < 16 years (the denominator) for each SA4 area | Residents 0-15 years sub-divided by SA4 | The level for each indicator was presented by   - statistical areas 4 (SA4) |
| Admitted Patient Data Collection (APDC) | Rate of hospital admissions for   - injuries (1-4 year-olds) - mental health conditions (13-17 year olds) - preventable conditions (5-12 year-olds)   calculated as the number of all hospital admissions for the specific health conditions (the numerator) being divided by the number of children of a specific age group in each SA4 area | Residents <18 years sub-divided by age and SA4 | The level for each indicator was presented by   - Australian Bureau of Statistics (ABS) Socioeconomic Indexes for Areas disadvantage quintiles - statistical areas 4 (SA4) |
| Australian version of the Early Development Instrument (AvEDI) | Proportion of children assessed as developmentally high risk (vulnerable in two or more domains including physical health and well-being, emotional maturity, communication skills and general knowledge, language and cognitive skills (numeracy and literacy); and social competence) | All children assessed, excluding those with special needs sub-divided by SA4 | The level for each indicator was presented by   - statistical areas 4 (SA4) |

^1^ The SA4 regions are the largest sub-State regions in the Main Structure of the Australian Statistical Geography Standard (ASGS) and have been designed for the output of a variety of regional data, including data from the 2016 Census of Population and Housing. They are specifically designed for the output of ABS Labour Force Survey data and therefore have population limits imposed by the Labour Force Survey sample. These areas represent labour markets or groups of labour markets within each State and Territory. A minimum of 100,000 persons was set for the SA4s, although there are some exceptions to this. In regional areas, SA4s tend to have populations closer to the minimum (100,000 - 300,000). In metropolitan areas, the SA4s tend to have larger populations (300,000 - 500,000). (source: <https://www.abs.gov.au/ausstats/abs@.nsf/Lookup/by%20Subject/1270.0.55.001~July%202016~Main%20Features~Statistical%20Area%20Level%204%20(SA4)~10016>)

^2^ These results represent crude rates only and may change after standardisation and spatial correlation adjustment. Figures below are some examples using currently available data.

Figure S1

The Western Australian Child Development Atlas example data visualisation maps


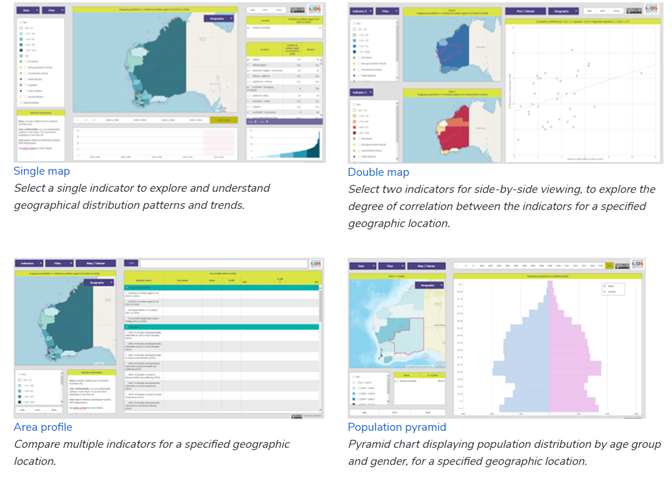


Figure S2

Children’s Health Queensland Population Health Intelligence Dashboard example data visualisation tools showing the regional proportion of children assessed to be developmentally vulnerable at school entry age using the AvEDI data by the levels of neighbourhood socioeconomic status (SES) measured using the SEIFA.


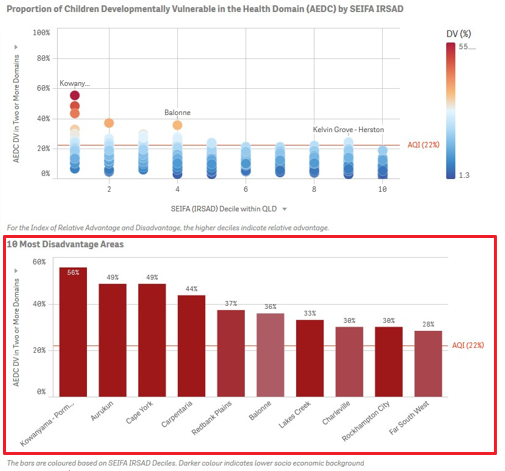


Figure S3

Children’s Health Queensland Population Health Intelligence Dashboard example data visualisation tools showing the positive correlation between two health indicators: low birthweight and maternal smoking during pregnancy.


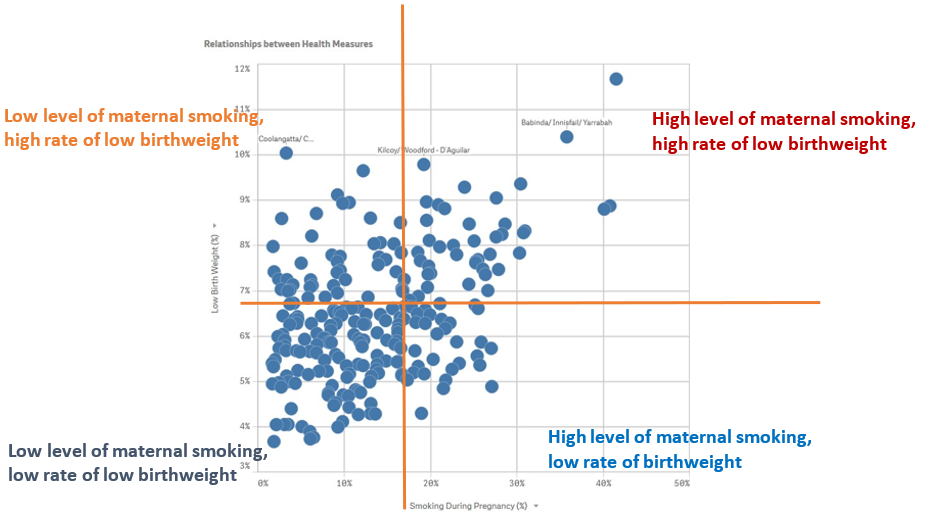

Supplement: sj-docx-1-him-10.1177_18333583231176597 – Supplemental material for Alpha NSW: What would it take to create a state-wide paediatric population-level learning health system? [file sj-docx-1-him-10.1177_18333583231176597.docx]
